# A theory-informed approach to developing visually mediated interventions to change behaviour using an asthma and physical activity intervention exemplar

**DOI:** 10.1186/s40814-016-0091-x

**Published:** 2016-08-15

**Authors:** Jennifer Murray, Brian Williams, Gaylor Hoskins, Silje Skar, John McGhee, Shaun Treweek, Falko F. Sniehotta, Aziz Sheikh, Gordon Brown, Suzanne Hagen, Linda Cameron, Claire Jones, Dylan Gauld

**Affiliations:** 1 Edinburgh Napier University, Sighthill Campus, Sighthill Court, Edinburgh, EH11 4BN UK; 2 Nursing, Midwifery and Allied Health Professions Research Unit, Unit 13 Scion House, Stirling Innovation Park, Stirling, FK9 4NF Scotland; 3 3D Visualisation Aesthetics Lab, Art & Design University of New South Wales, Sydney, Australia; 4 Health Services Research Unit, University of Aberdeen, Foresterhill, Aberdeen, AB25 2ZD Scotland; 5 Institute of Health & Society, Newcastle University, The Baddiley-Clark Building, Richardson Road, Newcastle upon Tyne, NE2 4AX England; 6 Fuse, the UK CRC Centre of Excellence for Translational Research in Public Health, NewCastle, UK; 7 Volunteer Centre Borders, First Floor, Riverside House, Ladhope Vale, Galashiels, TD1 1BT Scotland; 8 Asthma UK Scotland, Hayweight House, 4th Floor, 23 Lauriston Street, Edinburgh, EH3 9DQ Scotland; 9 Nursing, Midwifery and Allied Health Professions Research Unit, Glasgow Caledonian University, Cowcaddens Road, Glasgow, G4 0BA UK; 10 University of California, Merced, 5200 N. Lake Road, Merced, CA 95343 USA; 11 Health Informatics Centre, University of Dundee, Dundee, DD1 4HN Scotland; 12 Duncan of Jordanstone College of Art and Design, University of Dundee, Dundee, Scotland

**Keywords:** Intervention development, Asthma, 3D animation, 3D computer animation, Mixed-methods, Interdisciplinary, Visual

## Abstract

**Background:**

Visualisation techniques are used in a range of healthcare interventions. However, these frequently lack a coherent rationale or clear theoretical basis. This lack of definition and explicit targeting of the underlying mechanisms may impede the success of and evaluation of the intervention. We describe the theoretical development, deployment, and pilot evaluation, of a complex visually mediated behavioural intervention. The exemplar intervention focused on increasing physical activity among young people with asthma. We employed an explicit five-stage development model, which was actively supported by a consultative user group. The developmental stages involved establishing the theoretical basis, establishing a narrative structure, visual rendering, checking interpretation, and pilot testing. We conducted in-depth interviews and focus groups during early development and checking, followed by an online experiment for pilot testing. A total of 91 individuals, including young people with asthma, parents, teachers, and health professionals, were involved in development and testing.

**Results:**

Our final intervention consisted of two components: (1) an interactive 3D computer animation to create intentions and (2) an action plan and volitional help sheet to promote the translation of intentions to behaviour. Theory was mediated throughout by visual and audio forms. The intervention was regarded as highly acceptable, engaging, and meaningful by all stakeholders. The perceived impact on asthma understanding and intentions was reported positively, with most individuals saying that the 3D computer animation had either clarified a range of issues or made them more real. Our five-stage model underpinned by extensive consultation worked well and is presented as a framework to support explicit decision-making for others developing theory informed visually mediated interventions.

**Conclusions:**

We have demonstrated the ability to develop theory-based visually mediated behavioural interventions. However, attention needs to be paid to the potential ambiguity associated with images and thus the concept of visual literacy among patients. Our revised model may be helpful as a guide to aid development, acceptability, and ultimately effectiveness.

## Background

Visualisation techniques are increasingly being employed in an array of healthcare interventions [1]. However, the use of the visual can too easily be seen as an essentially inert media and simply a channel for a message that adds to uptake and engagement. This runs against growing evidence which suggests that appropriate choice of visual media can significantly enhance effectiveness [2]. The assumption that the visual is simply an inert channel means that the development of such interventions frequently fail to draw on sound theoretical [2] or design frameworks [1], with investigators often preferring a more inductive approach.

The success of a visual intervention may therefore be inhibited by poor operational definitions, lack of targeting underlying mechanisms upon which the intervention is (or should be) based, and poor evaluative planning. Through developing a strong theoretical framework, defining relevant concepts and constructs, and pre-defining evaluation methods, the development and evaluation of visual interventions can be strengthened. To move away from wholly intuitive creation of visual interventions, a four-stage process for development and evaluation was previously proposed [1], based on the UK Medical Research Council’s (MRC) Framework for Development and Evaluation of Complex Interventions [3]. This involved establishing a theoretical basis, modelling the structure, modelling the “look,” and checking interpretation and potential impact. Our research extends and refines this process to five stages, and we describe the refined and extended model stage in detail.

The current paper describes the theoretical and practical development and pilot evaluation of a complex behavioural intervention that sought to use visual media to mediate the theoretical basis and mechanism of action. The exemplar intervention focused on increasing physical activity among young people with asthma. Engagement in activity can lead to improvements in aerobic fitness [4, 5] and asthma-related benefits such as reduced hospital admissions, reduced absenteeism from school, fewer consultations with health professionals, reduced medication use [6], and improved ability to cope with asthma [5]. Children and adolescents with asthma should be encouraged to participate in regular physical activity [6], and they can exercise safely if appropriately treated [7]. Despite this, children and young people with asthma are less likely to be physically active than their peers [8–12], attributing this to their asthma. There is therefore an urgent need to develop novel strategies that address the specific barriers faced by young people with asthma in order to increase physical activity and exercise.

We therefore developed a visually mediated intervention to increase uptake and ongoing engagement in physical activity among inactive young people with asthma aged 12–18 years. The intervention had two sequential components, which aligned to and extended previously developed theoretical models for developing visual interventions [1]:

- An interactive educational component with motivational and skill developing elements that would create intentions to engage in increased activity.
- A child-specific activity plan agreed between parents, health professionals, and relevant school staff, and which would facilitate the translation of intentions into changes in behaviour.

This paper will present and discuss the explicit, novel, and theory-informed process through which the intervention was developed and the pilot evaluation method, with the aim of supporting others in developing similar visually mediated interventions; it will not discuss in detail the outcomes of the evaluation as this is outwith the scope of the current paper.

## Methods

The study was approved by the West of Scotland Research Ethics Committee (REC 4; Ref: 13/WS/0079). Appropriate NHS Research and Development and University ethics approvals were obtained. Participants provided written informed consent.

The methodology builds on previously successful theory-embedded animation work and associated model for intervention development [1] and goal setting/activity plan research [13, 14]. While the original four-stage model [1] had worked well, reflection on the earlier animation development suggested that alterations were needed. First, visual interventions should be underpinned with a substantive consultative base across all stages, including both future recipients and those who would have the responsibility for adoption and implementation in practice. With this in mind, we created a “consultative user group” (CUG). This group was recruited at the outset of the project and were involved in all subsequent stages, thereby providing a consultative structure and grounding for intervention development. Second, the modelling process should lead to a more formal experimental pilot testing stage of some of the internal mechanisms, so as to address any final changes to the intervention itself. The revised stages of development for visual interventions that we propose are:

- Pre-development stage—recruitment of an online consultative user group (CUG)
- Stage 1: establishing the theoretical basis—creation of conceptual content
- Stage 2: modelling structure—creating a visual narrative
- Stage 3: modelling the “look”—visual rendering of narrative and concepts
- Stage 4: modelling-checking—establishing interpretation and acceptability
- Stage 5: pilot experimental testing—exploring potential impact

**Fig. 1:**
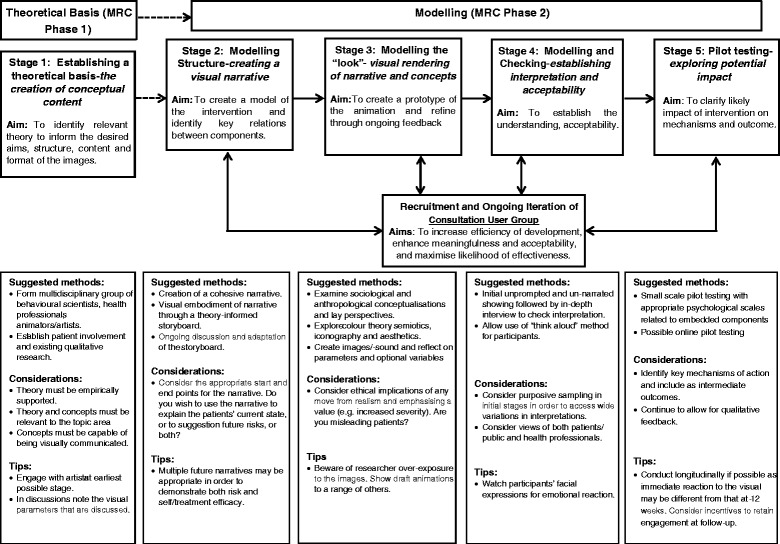
Revised five-stage visual intervention development model

The revised model proposed within this paper, and drawn upon for the development of the exemplar intervention, is shown in Fig. 1.

Note the key differences between the model proposed in Fig. 1 and that proposed by Williams and colleagues [1]: first, the addition of and continued involvement of a CUG across stages 2–5 and second, the addition of an explicitly stated and planned pilot testing phase.

### Pre-development stage—recruitment of an online consultative user group

To capture the diverse views of potential users of the 3D computer animation, we recruited a CUG. This ensured that the focus of the 3D computer animation was not overly “academic” or too theory-driven but instead encouraged human centred design through consistent feedback throughout the design process in key decisions (e.g. the look of the 3D computer animation, character design, physical activity selection for use in the 3D computer animation, feedback on the narratives, and storyboards). This increased the 3D computer animation’s acceptability and resonance with the intended user group, and the inclusion of the CUG was highly informative in shaping the 3D computer animation.

### Recruitment and membership

The CUG were recruited using a purposeful online recruitment, via asthma-related Facebook pages, forum groups, and other social networking sites. Snowballing was encouraged to recruit sufficient members. The CUG consisted of 23 people (six young people with asthma aged between 12 and 18 years, five parents (one father/four mothers), three teachers (one male/two females), six health professionals (all female), and three adults with asthma aged between 21 and 26 years (all female). The group was recruited online for practical reasons. The research team were located across different countries and institutions, and coordination across the research team and user group for face-to-face meetings would have been extremely difficult and potentially frustrating for CUG members. Having online recruitment also allowed us to recruit members from a wider population than those only able to attend meetings close to the host institution’s relatively rural location. Other studies may consider local, in-person contact to be more suited to their projects.

### Consultation mechanisms

The CUG were contacted via email and “message board alerts” linked to a private website and forum which only the Group had access to. Parents of young people in the CUG were asked to consent, and the parental email address was used to allow the parents full access to the group throughout the process. The research team shared and discussed ideas, useful resources, and design decisions through the use of a private online blog.

### Contribution to project decision-making

The CUG was recruited shortly after the project team was established and the overarching aims of the project had been established. Recruiting post-initial decisions allows for some pre-foci of the research aims to be established (and for recruitment of research staff and “settling” of a new research team to occur), thereby reducing wasted time or a lack of focus at the early stages of communicating with the CUG. A broad table of the involvement of the CUG within the project and their contribution to the project’s decisions is shown in Table 1.

**Table 1:** CUG involvement in the project

Stage	Project month	Key tasks relating to CUG involvement
Pre-development stage ---- Stage 1	1	1. Establishing team
		2. Discussing aims
	2	1. Defining and establishing aims
		2. Identifying potential theory base
		3. Identifying target user group and other related stakeholders
	3	Animation:
		1. Define theory
		2. Recruit CUG members
		2a) Welcome and introduce to team and online discussion forum mechanisms
		2b) Identify why interested/motivated for project
		2c) Set expectations of project
		Activity plan:
		1. Define theory
Stage 1 ---- Stage 2 ---- Stage 3	4	Animation:
		1. Storyboard iteration 1
		2. Moodboards
		3. Seek research team and CUG feedback to identify the “look”
		4. CUG discussion based on game theory on type and level of interactivity
		wIME pilot evaluation:
		1. Identify key themes and theory to be embedded in animation and the wIME
	5	Animation:
		1. Pre-visualisation of the look based on CUG feedback
		2. Iteration 2 of look—CUG feedback sought
		Activity plan:
		1. CUG discussion on facilitators and barriers to activity-informing narrative
		wIME pilot evaluation:
		1. Finalise key themes and theories
Stage 2 ---- Stage 4	6	Animation:
		1. Discuss with CUG the kinds of activities to be used in the animation, using public forum threads to facilitate and inform the discussion too
		wIME pilot evaluation:
		1. Used CUG discussions on facilitators and barriers to inform vignette design for pilot evaluation
Stage 3 ---- Stage 4	7	Animation:
		1. Character artist develop eight prototype characters. Feedback from CUG and online survey. Changes made to characters based on feedback and least popular ones removed.
		2. Three activities for inclusion in animation selected based on CUG discussion.
		wIME pilot evaluation:
		1. Develop first iteration vignettes and seek CUG feedback.
Stage 3	8	Animation:
		1. Revise characters
		2. Create background settings for animation
		3. Begin building 3D animation elements
		wIME pilot evaluation:
		1. Embed theory in second iteration of vignettes
Stage 2 ---- Stage 3	9	Animation:
		1. Develop narrative, audio, and visual storyboards. Seek CUG feedback.
		wIME pilot evaluation:
		1. Identify questions for pilot evaluation
		2. Submit ethical application
Stage 2 ---- Stage 3 ---- Stage 4	10	Animation:
		1. Iteration 2 of narratives
		2. Integrate storyboards into animation.
		3. CUG feedback on narrative and look
	11–13	Animation:
		1. Integration of feedback and continued development of animation
Stage 1 ---- Stage 4	14	Animation:
		1. Feedback on usability
		Action plan:
		1. Key theories identified
		2. Content of action plan identified
		3. Seek CUG feedback
Stage 4 ---- Stage 5	15	Animation:
		1. Final iteration and pilot evaluation begins
		Action plan:
		1. Integrate feedback to develop second iteration
		2. Seek feedback
Stage 2 ---- Stage 4 ---- Stage 5	16–18	Animation and wIME:
		1. Pilot evaluation active
		2. Updating CUG on pilot testing progress monthly
		Activity plan:
		1. CUG feedback on activity plan
		2. Final iteration of activity plan
Stage 5	19	1. Analysis
		2. CUG feedback on interpretation of data
		3. Final debriefing with CUG

Projects employing a CUG should carefully consider the level of involvement required for a CUG, and maintenance of communication throughout the project with the CUG, even when no specific task/feedback is required of them. The purpose of continued communication is twofold: first, to maintain engagement with the project and avoiding drop out due to lack of interest via lack of communication; second, out of respect for the time and engagement offered by the CUG members—even though there may not be a task, maintaining communication about project updates allows the CUG to become and feel like a part of the research team (as, of course, they are). Levels of engagement should be discussed at the outset of the project.

Not all of the CUG members participated in all of the discussions nor were they required to. All members did participate in at least two discussions, but it was acknowledged that there would be times over the project when availability would be limited (e.g. exam periods for the young people) and so feedback and participation was wholly voluntary.

### Stage 1: establishing the theoretical basis: creation of conceptual content

#### Literature review

**Fig. 2:**
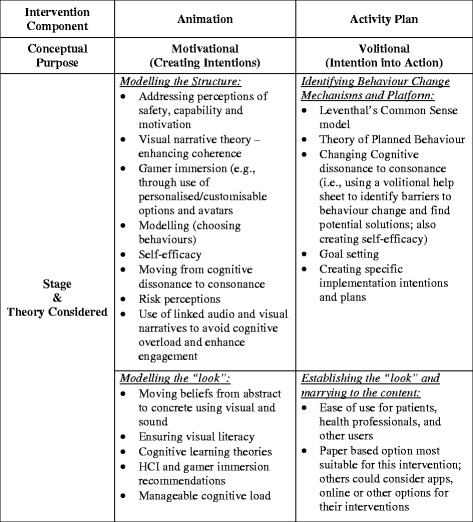
Theory and theoretical basis for the intervention and integration in development

Williams et al.’s [1] earlier recommendations for developing a theoretical basis for visual interventions indicated three criteria: first, sufficient evidence must exist in prior empirical research to suggest that the theory could lead to behaviour change; second, the theoretical concepts must be operationally relevant to the relevant population; and third, the theory must be visually communicable so as to be embedded within the visual intervention. This is not to say, however, that non-visually communicable/operational theories should not be considered in the broader scope of behaviour change interventions but should be carefully considered if using in purely or mainly visual interventions. In addition, broad behavioural approaches suggest that consideration is given to both addressing *motivational* issues in order to create intentions and a *volitional* stage that enabled these intentions to be translated into behaviour. Figure 2 shows the theory and theoretical rationale underpinning both elements of the intervention, along with an indication of the stages at which they were included. While there was overlap in considerations about the theoretical models underpinning the whole intervention (i.e. the behaviour change models considered), Fig. 2 highlights the most influential underpinning concepts for each component. The concepts identified for the current intervention may not be suitable for all visual interventions, and so literature review, discussions, and considerations for what is needed should be considered on an intervention-to-intervention basis. We present the underpinnings outlined in Fig. 2 as part of our exemplar and a reasonable set of considerations for other, similar interventions.

In accordance with the MRC Framework for Complex Interventions, our theoretical basis drew on both primary research and existing theory [3]. Barriers and facilitators to engagement in physical activity had already been identified in an in-depth study of children’s, parents’, and school staff views and experiences [15–17]. This study identified factors influencing participation in physical activity and also uncovered the illness beliefs of young people, parental and family beliefs about both activity and asthma, and the knowledge and attitudes of teachers and the organisational arrangements in schools and provided insight into children’s, parents’, and teachers’ beliefs about *capability*, *motivation*, and *safety* in relation to activity levels among children with asthma. We therefore sought to address these key factors, i.e. (1) beliefs that it might not be safe to exercise (risk perception), (2) beliefs that they would not be capable of exercising (self-efficacy), and (3) motivation (not interested in types of exercise suggested) within the design of the intervention.

While these core issues were at the heart of what the intervention aimed to challenge, further work was necessary in order to identify relevant theory that might inform the mechanism by which such beliefs could be modified and thus lead to behaviour change. This was the result of a broad-scope review of the literature and “round-table” research team (experts in the area of complex visual intervention development for behaviour change) discussion and deliberation. The literature review yielded 119 papers of direct relevance to the current project, from which we extracted eight key themes: visual narrative theory, interactive multimedia development, games narrative/storyline development, cognitive dissonance/consonance, health and behaviour change models, asthma experiences, gamer immersion, and cognitive ageing/learning. A recurrent subtheme across the behaviour change, asthma experiences, and gaming/learning literature was the importance of self-efficacy. This was therefore added as an important cross-cutting theme in and of itself to the existing list. These themes were summarised by one researcher and discussed by the whole team.

### Developing an activity plan

A recent meta-analysis has established that creating specific implementation intentions (i.e. forming an implementation intention that spells out the when, where, and how of goal-striving in advance) increases the likelihood of behaviour change [14]. The interactive 3D computer animation focussed on generating intentions alone. It was clear that evidence suggested that an additional intervention component, an action plan, would increase the likelihood that behaviour change would be achieved and sustained. In the context of asthma, this approach is supported by evidence showing the effectiveness of personalised asthma action plans [13].

The activity plan developed for the present study was adapted from work in other areas (e.g. [18, 19]) to promote physical activity in a population with asthma. Participants received a sheet of paper containing information about the importance of making the activity plans and open text boxes where they could fill in their own action plan. The developed plan consisted of two components: (1) an action plan, asking young people to specify when, where, and how they would engage in physical activity and (2) a volitional help sheet, which asked the young person to identify potential barriers to their engagement in physical activity and how they might overcome them (copies of this are available from the corresponding author).

### Stage 2: modelling structure: creating a visual narrative

Once the theoretical basis for the intervention had been established, it was necessary to embed it in a framework that would underpin and provide structure to the intervention itself. This “framework” took the form of a narrative that enabled logical links between the theoretical components and the outcomes, while simultaneously engaging the participant. Narrative has a history within sociological studies of patients’ experience and understanding of illness [20–22]; however, it also has increasing recognition within the psychological literature [23, 24]. It can facilitate attention and engagement, aid memory [25], and provide a means by which causal links between sequential events are understood [26]. Past work on behavioural modelling [27] has illustrated how self-efficacy [28] and knowledge can be increased [29].

Thoughtful development of characters has the potential to add to the power of a narrative [30]. Creating an empathic bond with a character should, theoretically, increase user attention and engagement with the 3D computer animation [31], increasing immersion in the narrative [32]. This principle is increasingly employed within the game industry through the ability of the individual to choose and create their own character, and thus create an optimal learning environment in which to focus on key messages.

### Process of narrative construction

To create a narrative that embedded our underlying theory, and enable clear discussion and consultation among research team members and the CUG, we adopted the previously successful concept of the “theory-embedded storyboard”. Storyboards are used by designers to provide a common visual language that people from different backgrounds can understand and discuss prior to full rendering [33].

An initial narrative was jointly constructed by the team. At each of the key stages of design, members of the research team reviewed whether the theory was clearly embedded all aspects of the visuals. Where uncertainties arose within the team, the CUG and/or other members of the research team were consulted. Aspects of the theory that were considered central to the 3D computer animation were further evaluated in the piloting of the intervention (described later). The second step involved developing the audio narrative to support the visual aspects of the 3D computer animation. The audio was written as the character’s “first person” account of their asthma experience and was conversational in tone. This was important to build rapport and empathy [31], to increase engagement and investment in the character [32]; thereby increasing learning [34]. The information contained key safety, motivational and self-efficacy building statements [1]. In addition, and in line with Williams et al. [1], the inclusion of sound to emphasise breathing and wheezing was used within the audio narrative. The reason for this inclusion was twofold: first, to create a sense of urgency [35] in the wheezing micro-narrative and, second, to convey meaning beyond the use of words [36].

### Stage 3: modelling the “look”: visual rendering of narrative and concepts

Although decisions concerning visual rendering are frequently taken within a range of commercial industries in order to achieve cognitive engagement, emotional representation, and aesthetic appreciation, there is relatively little formal empirical research available to inform decision-making. However, visual appearance does have the potential to make a difference in regard to behaviour change—particularly in the ability of different visual forms to fully convey key theoretical principles and mechanisms that have been chosen. For example, recent analyses of the impact and components of the visual warnings on UK cigarette packs have shown that the choice of visual form—ranging from abstract (e.g. cartoon), through representation (e.g. X-ray image), to concrete realism (e.g. photograph)—appears to have an effect on intentions and emotions after having controlled for other factors [37, 38].

### Process of visual rendering

The practical development of the interactive 3D computer animation, including embedding the theoretical basis, was an iterative process, involving the formation of a sub-team of researchers. This sub-team included two 3D computer artists, a psychologist, a sociologist, and an asthma nurse involved in applied intervention development and evaluation research. All members of the sub-team were involved in key decisions about the 3D computer animation’s design and development. The team met monthly and communicated via two private “blogs” in between these meetings. In addition to the sub-team meetings, quarterly meetings were held which involved the entire research team. A character animator was also hired and involved in the early stages of the 3D computer animation design specifically to create the “look” of the characters and backgrounds used. The composition and management of the project team, including regular face-to-face and online means of communication was the key to keeping the momentum of the development on track and in creating the 3D computer animation with the theoretical basis embedded as effectively as possible.

### Stage 4: modelling-checking: establishing interpretation and acceptability

Although development of the intervention had a sound theoretical basis and was formed in consultation with a range of stakeholders, this would not guarantee its acceptability and meaningfulness to the relevant target audience. Consequently, the final developmental process focussed on addressing two aims:

1. Assess the understanding of and acceptability of the intervention as a whole by the key stakeholder groups (young people with asthma, their parents, health professionals, and school teachers).
2. Identify any further changes required to one or more elements of the 3D computer animation required modification—what worked and what did not.

In order to address these aims, we conducted in-depth qualitative interviews with young people, parents, and health professionals.

### Sampling and recruitment

We intended to recruit five young people (aged 12–18 years with active asthma), five parents and five health professionals for early feedback through primary care, and then later recruit ten young people with asthma and their parents to show the full 3D computer animation. However, we experienced significant recruitment problems (i.e. lack of responses to our recruitment adverts and direct requests for participation). This was unanticipated for three reasons: we employed methods that we had found successful in previous primary care studies that we have conducted over the past 20 years; there was very little input needed from the primary care team; and we included GPs and practice nurses in the research team with intimate knowledge of what is usually acceptable and effective. Despite this, recruitment was difficult. We approached 60 practices by post and telephoned all practices (some on several occasions). After an initially disappointing response to phone calls, we used a second researcher and health professional with skills known to be excellent in recruitment. We made two ethics amendments to adapt recruitment and finally extended recruitment into secondary care asthma clinics. In response, we drew more heavily on the established CUG, as this included 23 individuals from the target groups (young people with asthma, parents, health professionals, and teachers). Our final consultation and feedback numbers are shown in Table 2. While the target sample was not met and recruitment was an issue, we chose to adapt our intended methods to allow the continued development and evaluation of our intervention. This experience is pertinent to designers of similar interventions, as it demonstrates the need to be reflexive to research design modifications.

**Table 2:** Consultation numbers and methods for iterative development

	Young people with asthma	Adults with asthma	Parents	Health professionals	Teachers	Total
Consultative group (CUG)	5	3	4	5	6	23
Interviews/focus groups	4	0	3	4	4	15
wIME	29 (12)^a^	0	24 (14)^a^	0	0	53
Total	38	3	31	9	10	91

^a^Number in parentheses indicates number participating at follow-up

### Data collection

Participants (young people with asthma, parents, health professionals, and teachers; see Table 2) were asked to arrive 45 min before their asthma review in order to view the 3D computer animation, make an activity plan, and be interviewed. Participants were asked to use the “think-aloud” technique (i.e. say out loud what they thought about when they viewed the 3D computer animation [39]). A semi-structured interview followed immediately after viewing the 3D computer animation. Participants were asked about their thoughts on the intervention as a whole and what they thought of the different sections. Two focus groups were also held: one with teachers and one with health professionals.

### Analysis

Interviews were recorded, transcribed, and analysed drawing on the Framework Method ([40]; see also Appendix 4). Analysis was conducted by SS and BW. Given that the purpose of the interviews was to check meaning rather than to inductively explore and conceptualise pre-existing view and experiences around asthma, analysis at a descriptive rather than conceptual level was regarded as most appropriate.

## Results

The qualitative findings suggested six themes: perceived initial impact, asthma understanding, relevance to self, intentions, suggested improvements/amendments, and perceived clinical appropriateness/usefulness. Supportive quotes across these themes are shown in Appendix 1. Feedback was positive from all groups, with the volume and complexity of information presented being regarded as acceptable, particularly as the individual was able to interact with the 3D computer animation and therefore run through different inhaler taking scenarios. The perceived impact on asthma understanding and intentions was reported positively, with most individuals saying that the 3D computer animation had either clarified a range of issues or made them more real. Parents, teachers, and health professionals all felt that the 3D computer animation would be a particularly engaging and useful tool. A number of suggestions for improvement were made. These focussed predominantly on expanding the character selection and the activity options. Participants felt that an increased number of characters of different races would be beneficial to different racial groups using the animation. They also felt that having more options for activity selections may be desirable, including swimming or other team sports. This would require, however, several months of additional work for the graphic design and animation team.

### Stage 5: pilot experimental testing—exploring potential impact

At this stage, we did not seek definitive evidence of effectiveness. Rather, we were concerned with assessing potential impact on intervening variables. We conducted a web-based Intervention Modelling Experiment (wIME) [41]. The wIME was hosted on LifeGuide (www.lifeguideonline.org). Participants were recruited online, via social networking sites (i.e. Facebook (a full list of the groups targeted is included in Appendix 2), Twitter) and through specific online asthma-interest groups (i.e. Asthma UK fora, Asthma UK emailing list, PsychPostgrads emailing list, direct emails to health professionals). Snowballing was encouraged: participants were asked to distribute the link to the questionnaire to potentially interested parties. Target participants were young people with asthma (12–18 years) or parents of young people with asthma.

Participants completed a baseline questionnaire, were randomised to receive the intervention or control, and were then requested to complete the same questionnaire 2 weeks later. Data were collected on asthma severity, activity level, illness beliefs [42], questions drawn from the theory of planned behaviour [43], and risk/worry about asthma. Current levels of physical activity, intentions to engage in physical activity, inhaler use, and finally, reactions to specific vignettes relating to projected inhaler use and physical activity decisions were measured. We also piloted a range of behavioural scenarios to examine whether behavioural intentions could be assessed and measured. These were created jointly by the research team, health professionals, and CUG and were developed in line with empirical guidance on effective vignette development ([44, 45]; see Appendix 3). They presented a range of situations that young people might find themselves in with regard to their asthma symptoms and physical activity and asked them to express their behavioural intentions in these situations. Fifty-three participants completed the baseline questionnaire (24 parents, 29 young people). Twenty-six completed both the baseline and the second sitting of the questionnaire (14 parents, 12 young people). A full discussion of the evaluation findings will not be presented here; these can be found elsewhere [46]. The current paper aims to only highlight the process through which we developed the exemplar and propose a forma pilot testing stage as a key component to developing visually mediated interventions.

### Final intervention

Our final intervention consisted of two components—an interactive 3D computer animation to create intentions and an action plan and volitional help sheet to promote the translation of intentions to behaviour.

**Fig. 3:**
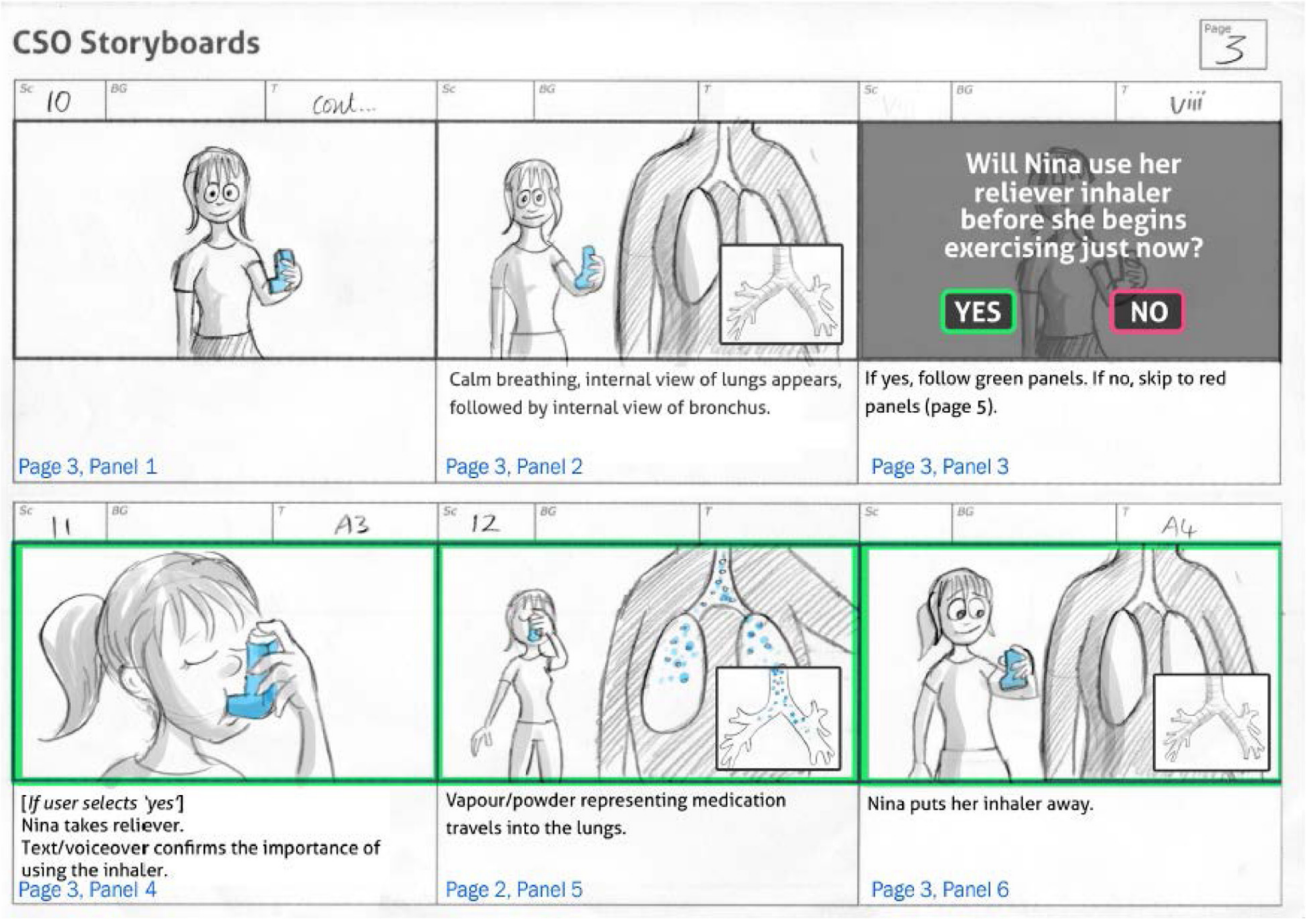
Exemplar storyboard: building the narrative

**Fig. 4:**
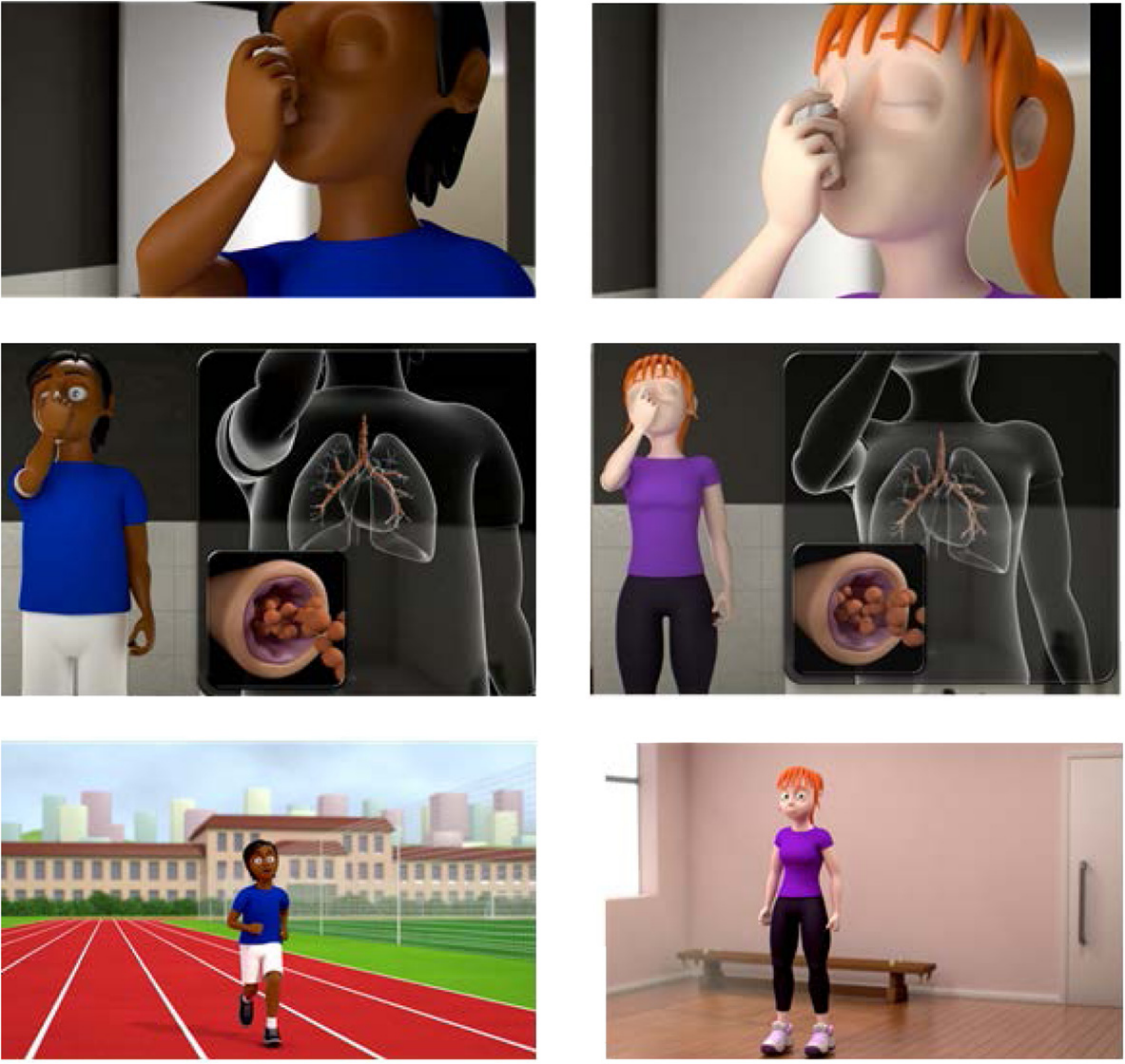
Exemplar visual rendering

The final 3D computer animation allowed participants to interact with it and explore choices that their character made. First, they decided which character they would like to use. The characters consisted of a male and female of differing ethnicities. Both characters were dressed in “sporty” but not “trendy” outfits and were designed to look between 12 and 18 years old. They then choose which type of activity their character would engage in dancing or running. The character was then introduced, and the decision is made by the user whether or not they would take their preventer inhaler. An explanation of asthma and the action of preventer and alleviator inhalers followed, with visual close-ups of the lungs to show how they work and are beneficial. The sound of breathing—both clear and laboured as appropriate—was also used. They then moved onto taking part in the physical activity and became breathless. The choice whether to take the reliever inhaler was then made, and the character either carried on with their activity (if inhaler was taken) or was visibly impeded and had to stop and take the inhaler. In both cases, the character was able to resume physical activity, with the audio narrative directing the participant to messages relating to safety and self-efficacy (e.g. “you can exercise safely, with adequate monitoring of your condition and using inhalers”). A final screen was shown with these messages in text, and the 3D computer animation was complete. The user then had the option to return and make different decisions, change character, change activity, or end the 3D computer animation. The 3D computer animation lasted approximately 7 min, depending on which micro-narratives were selected. A selection of screen captures to illustrate sections of the narrative is shown in Fig. 3 and exemplar rendering in Fig. 4.

Figure 3 illustrates captures of each character taking their inhaler, the external-internal visualisation of the medication (abstract to concrete visualisation) and an example of the characters in the different physical activity backgrounds (running track and gym hall). Figure 4 shows an example of the storyboard used to develop and integrate the visual and audio narratives and to allow team and CUG discussions.

The final activity plan was paper-based for ease of use across different settings. It consisted of two A4 pages: an action plan and a volitional help sheet. The action plan (page one) contained information about the importance of making an action plan to motivate physical activity and asked users to complete three open text boxes specifying when, where, and how they would engage in physical activity. The volitional help sheet (page two) provided examples of barriers to physical activity and provided example solutions to these. It asked users to identify their own potential barriers to their engagement in physical activity and how they might overcome these.

## Discussion

### A revised model for the development of visually mediated behavioural interventions

Our study has demonstrated that, with care, detailed consultation and a willingness to explore theory and evidence from diverse academic and non-academic sources, it is possible to translate key behavioural concepts which are routinely embedded in behavioural interventions, into visual forms. Furthermore, these visual forms may prove highly acceptable and engaging among a target audience. There are good reasons to believe that visually mediated interventions may become increasingly common and important within healthcare making such detailed approaches more important.

Healthcare providers are increasingly using explicit imagery through public advertisements to promote not only health behaviour but also altruistic acts in the form of blood and organ donation [47]. The proposed social shift towards a more visually based society might support this. In addition, more recent research has suggested that individuals may differ in terms of their position along a “verbaliser”–“visualiser” continuum [48], with even more recent work suggesting that “visualisers” themselves may vary in type [49] and ways in which information is processed [50]. With this in mind, it may be that the medium through which a behavioural intervention is delivered may not be neutral or innocuous but may also act as either a facilitator or detractor to the meaning and effectiveness of that intervention.

Our detailed and iterative approach to intervention design, with consultation and meaning checking built in throughout, led to an accurate interpretation by participants. However, with less care and attention, images may have the potential to cause confusion or misinterpretation. Different individuals will interpret the same images in different ways thereby leaving room for potentially problematic ambiguity. While the concept of visual literacy has begun to gain recognition, and suggests that there may be common symbols that societies use to communicate meaning, it also suggests that people vary in their interpretive abilities [51, 52]. In practice, this means that both the development and methodological evaluation of visual interventions must assess correct interpretation and even tailor images according to different subgroups. This process might best be addressed through what the MRC Framework for Complex Interventions terms the “modelling processes” of intervention development. Our study suggests that such modelling may benefit from drawing on sophisticated online means of presenting images with varied parameters and assessing meanings across different populations, but also that recruitment of a user group that can respond rapidly to detailed decisions about visual forms and structure may be of great value. With these lessons in mind we suggest that the revision to our original model [1] for the development of visually mediated interventions be used (see Fig. 1). We do not suggest that this be prescriptive, but could be used to guide decision-making to maximise both acceptability and effectiveness, while minimising the chances of misinterpretation and harm through ambiguous image and narrative.

### Theory and mechanisms of impact for visually mediated interventions

We identified a theoretical basis for our intervention a priori, participants’ description of the 3D computer animation as “bringing it home” suggested that another mechanism might be operating through the 3D computer animation in order to motivate behaviour change. Conceptually, this issue may be accounted for through Leventhal’s suggestion that illness representations may be characterised as lying on a continuum from abstract/conceptual to concrete/experiential [53, 54] and as representations that are increasingly concrete have the potential for greater impact. Visual animation may move such representations from abstract knowledge to more concrete experience, particularly where sound is also employed. This theory also concurs with work in semiotics which suggests that communication may be deconstructed into “modality” (the experiential form of the object or concept being communicated, e.g. visual, auditory, tactile, olfactory, gustatory, kinesthetic) and “medium” (the experiential form through which the thing is communicated, e.g. text, speech, sound, visual). A message may employ multiple modalities. The more of these modalities that are embodied via the medium then the greater the “sense of reality” that will be achieved [55]. This requirement for sensory communication and physical coherence is paralleled within the computer graphics and arts disciplines and seen as a key to achieving what has been termed “presence”, the sense that what may be distant or in fact a virtual representation is actually somehow present and real [56]. Our decision, therefore to include the sound of breathing and wheezing is likely to have contributed to moving observers’ illness representations from the abstract to the concrete and therefore, while perhaps not increasing knowledge per se, instead simply “bringing it home”.

The suggestion that engagement of the senses can move beliefs from the abstract to the concrete suggests that further consideration could be given to behavioural interventions which are more fully sensory than the sight and sound embodied in our intervention. Indeed, in some areas of healthcare, this is already taking place. For example, infant simulators have been introduced to “bring home” the reality of early motherhood among teenagers [57]. Again, the methodological development of such “sensory-mediated” interventions would require significant attention. Current recommendations and guidance with regard to how such interventions should be developed, how theory could be embodied, and what techniques might best be employed to assess their impact are currently lacking.

## Conclusion

We believe that given an increasing social preference for visual forms of communication, there is a need to develop the evidence base and identify relevant theory to support behavioural interventions that use such media. We have demonstrated the ability to develop theory-based visually mediated behavioural interventions. However, attention needs to be paid to the potential ambiguity of images and thus the concept of visual literacy among patients. Our revised model may be helpful as a guide to aid development, acceptability, and, ultimately, potential impact. The research team plan to carry on to conduct feasibility trials and a randomised control trial to identify whether and where the intervention can be feasibly used in asthma care (e.g. primary care, secondary care, schools) and whether it actually evokes behaviour change.

## Abbreviations

CUG, consultative user group; wIME, web-based intervention modelling experiment

## Appendix 1

**Table 3:** Qualitative data from initial piloting (*YP* young person, *P* parent, *T* teacher, *INT* interviewer)

Theme	Quote
Global impression/initial impact	YP: … it was easy to understand, yeah it was good.
	-----------------------------
	YP: I think it is all very clear. It does make sense.
	-----------------------------
	YP: It was good. Yeah it was good……. Taught you why to use it, well it didn’t really tell you how to use it, and it told you when to use it and when to
	------------------------------
	YP: It was good; it had a lot of information in it.
	INT: Yeah, was it too much?
	YP: No, honestly.
Perceived Impact on Asthma Understanding	P: And especially the bit where it showed the little capillaries inside the canister… cause I think for children… to see it, so I think that actually just puts it in their head that there is actually something in there… so I felt that was really good, especially for wee ones. Just cause… you know, cause he was quite young when he got it, so I think that was quite… maybe it was more age for maybe seven or eight I think or even smaller it could be even more simplified, but I think it’s very good.
	-------------------------
	INT: So how helpful did you find, in your own words, how helpful did you find the animation?
	YP: I found it quite helpful because it did explain to you well how you can do things if you have asthma, and even if you need to take your inhaler during it and you can still keep going.
	--------------------------
	YP: I think it (*animation*) has improved it (*knowledge of asthma*).
	INT: In what way?
	YP: What I know about asthma and what I think about it.
	--------------------------
	INT: was it too much information sometimes?
	YP: No, cause it maybe even better for more, like, what exactly happens, so it was very clear.
	P: I thought that was very good as well.
	--------------------------
	YP: … they described it well cause, like, it had the preventer and the reliever one, so it talked about the different ones, like, how they work.
	--------------------------
	YP: … like for me I knew all that already, but maybe someone that’s new to it, that would be helpful yeah, that would be helpful to someone that’s new.
	--------------------------
	T: What is actually happening in the body when that’s happening, I was interested to see that cause I didn’t know, just they had to take an inhaler but I know why.
	T: It was interesting to see that the reliever one, I presumed that they would use that after they started to feel… and it looked like it say no actually you take that before.
Perceived relevance to self	YP: When I saw the one with Ajay, he did the one activity that I also do.
	---------------------
	INT: Was it too much information for them?
	P: I don’t think for a 12 year old, it’s quite basic, it’s quite basic for Sarah’s age and maybe to 18, but for kids and stuff I think it’s good. It was explained, it simplified it down which was good and it showed you how… would work.
	---------------------
	INT: What did you think about the sound, you know, when he was a bit wheezy?
	YP: I sort of recognised it straight away.
	INT: Was it very life like?
	YP: Distinctive.
Perceived Impact on Intentions	INT: So would you consider being more physically active, but I guess you have to talk with your GP or your health professionals and things before that, but your view of physical activity and asthma, d’you find it difficult?
	YP: Yeah I find it quite difficult to do stuff like that but watching that, telling you what to do and stuff before sport and stuff.
	---------------------
	INT: Okay. And after viewing the animation, do you think you’re more motivated to be more physically active?
	P: Definitely.
	---------------------
	INT: So would you say you’re more motivated to encourage children to take part in physical activity even though they have asthma?
	T: Yeah, wouldn’t feel as awkward about approaching them, yeah, feel more confident.
	T: And I would say after, like I said, five minutes saying ‘come on then, come back in now, come and try again now’.
	T: Even more so I’d be more inclined to speak or have a chat more with whoever’s got asthma now and just understand a bit more about their own individual case.
Suggested Improvements	YP: Maybe include another activity, just something that you do in everyday life.
	----------------------
	YP: Maybe another character that you wouldn’t think about having asthma.
	----------------------
	T: Like swimming or something.
	----------------------
	T: And perhaps more emphasis on the benefits of exercise.
	----------------------
	P: I would’ve made it a blue inhaler cause that’s, like, quite a common one and everyone sort of has the blue one, so I would’ve made that blue, but that was it, everything else was fine.
	----------------------
	YP: And maybe one (character) that’s a bit heavier ‘cause if they’re on steroids then they do…
	----------------------
	P: Yeah, well if you added more activities, maybe some more characters, that would be it, it’d be fine, but like make it a blue inhaler, I would make it a blue inhaler or even, like, show two inhalers.
Perceived Clinical Appropriateness, Usefulness	T: Yeah, you could do it through a health and wellbeing, part of your topic type of thing as well, talking about benefits of exercise, everybody can take part in exercise and… asthma can take part, and then if someone’s going to take an inhaler they’re not like ‘oh what are they doing over there?’ they know, they understand it as well so it’s educating everybody, not just those with asthma.
	-----------------------
	T: No, I mean, if a class teacher saw this, say, once a year and you could say to them ‘listen, we’re doing a continual run or a fitness circuit for the next few blocks, definitely maybe make them bring them’. As I say, I’ve got lots of schools where the puffers are in an office, bring them so they’re here and, you know, some kids if they want to take it preventative, ‘cause they know more.

## Appendix 2

### List of groups targeted and contacted for participant recruitment and number of followers/recipients in each group (in parenthesis) at time of contact where identifiable

1. Asthma UK Beach to Beach Bike Ride Facebook page
2. Asthma UK Royal Parks Foundation Half Marathon Facebook page
3. Big Up Your Chest Facebook Page
4. Asthma UK London Marathon Team Facebook page
5. Asthma Facebook page
6. Healthtalkonline Facebook page
7. Asthma and Indoor Allergy Sufferers discussion page Facebook page
8. Asthma UK emailing list
9. PsychPostgrads emailing list (935 recipients)
10. Asthma UK Facebook page (> 9000 followers)
11. Asthma UK Three Peaks Challenge Facebook Page (132 followers)
12. Asthma UK Bupa Great North Run Facebook page (241 followers)
13. Asthma Challenges Facebook page (1801 followers)
14. Asthma Awareness Facebook page (959 followers)
15. Surviving Asthma Facebook page (356 followers)
16. Asthma SUCKS!!! Facebook page (729 followers)
17. Asthma Facebook page (63 followers)
18. Asthma Puffers Facebook page (79 followers)
19. World Asthma Foundation Facebook page (4768 followers)
20. Allergy & Asthma Network Mothers of Asthmatics Facebook page (1973 followers)
21. Asthma UK Loch to Loch Bike Ride Facebook page (546 followers)
22. Asthma UK Challenges Facebook Page (240 followers)

## Appendix 3

### Description of the vignette development for the wIME

*The vignettes were developed in line with literature on vignette development*:

Ashill, N. J., & Yavas, U. (2006). Vignette development: an exposition and illustration. *Innovative Marketing*, 2(1), 28–36.

Heverly, M. A., Fitt, D. X., & Newman, F. L. (1984). Constructing case vignettes for evaluating clinical judgment: an empirical model. *Evaluation and Program Planning*, **7**, 45–55.

*These papers propose that vignettes intended for empirical research should*:

1. Be of approximate equal lengths, preventing bias in terms of readers “skimming” the longer passages, or focusing in greater detail on longer or shorter passages due to difference in length thereby altering the level of attention/importance given to each.
2. Information provided in vignettes should be relevant to the study’s research questions and aims.
3. Information given across vignettes should be consistent (e.g., all should have names, ages, genders etc., this should not appear in some but not others). This will help to determine which cues are consistent across vignettes and which, if any depending on study design, should be manipulated.
4. Consistent information (see above point) should be shown in similar orders. This will prevent primacy/recency effects acting as an extraneous variable(s). That is, participants paying closer attention to information at the beginning (primacy) or end (recency) of the vignette. Ideally, if a large number of vignettes are being developed, this information should be counterbalanced. Manipulated cues/information should appear in approximately the same location throughout the vignettes, or should be counter balanced.
5. Vignettes, if being manipulated, should not be too long (more than half a type written page, size 12 font). This is to prevent extraneous variables/information/cues influencing the judgement made and to allow for successful manipulation (if too much additional information is included, the success of the manipulation is reduced—this is largely due to the large number of cues demanding attention and potentially detracting from the manipulated cues).

*Asthma and physical activity vignettes*:

- Sixteen vignettes were required: eight for the pre-intervention condition and eight for the post-intervention condition.
- We have an intervention (the animation) with two gender types, three race types, and three activity types. Lack of physical activity is also a focus, so we therefore have four activity types:
  1. Gender: male, female
  2. Race: White, West Asian, East Asian
  3. Activity: running, dance, football, no activity
- Gender and activity are important for the vignettes; race is not, as physical activity is important for all races and should not be seen to differ.
- We have two types of inhaler decision and two types of inhaler:
  1. Preventer and reliever
  2. Take inhaler versus do not take inhaler
- In terms of the animation, the reliever inhaler is the one where the user is given the choice to take or not, so we will only use this in the vignettes to reduce the volume of information per vignette and cross balance these.
- Our age group is 12–18 years old.
- We have two viewing conditions: pre- and post-intervention reading of vignettes.
- The post-intervention vignettes will need to carefully match the pre-intervention ones in terms of balancing information.

*Required content for each vignette and number of conditions/levels (in brackets)*:

- Name (N/A)
- Age (two conditions; younger/older)
- Gender (two conditions; male/female)
- Activity type (four conditions; none/running/rugby/dance)
- Take inhaler (two conditions; yes/no)
- Sixteen vignettes; eight pre- and eight post-intervention

*Vignette essential content: pre-intervention (1a-8a) and post-intervention (1b-8b)*:

**Table:**

Vignette code	Name	Pre-/post-intervention	Gender	Age group	Takes inhaler	Activity type
1a	John	Pre	M	Y	Y	Run
1b	Richard	Post	M	Y	Y	Run
2a	Sally	Pre	F	Y	N	None
2b	Jane	Post	F	Y	N	None
3a	Harry	Pre	M	Y	Y	Football
3b	Dylan	Post	M	Y	Y	Football
4a	Nicola	Pre	F	Y	N	Dance
4b	Anne	Post	F	Y	N	Dance
5a	Graham	Pre	M	O	Y	Dance
5b	Brian	Post	M	O	Y	Dance
6a	Mary	Pre	F	O	N	Football
6b	Elizabeth	Post	F	O	N	Football
7a	William	Pre	M	O	Y	None
7b	Josh	Post	M	O	Y	None
8a	Jasmine	Pre	F	O	N	Run
8b	Leona	Post	F	O	N	Run

## Appendix 4

### Description of framework analysis used for qualitative data [40]

Framework analysis is a systematic form of thematic analysis which uses clear steps for the analysis process and produces highly structured, summarised data; ideal for the current project’s aims. Using this form of analysis, the key themes and subthemes from the data were identified both within the individual focus groups/interviews and across the whole data set, providing a highly descriptive overview of the whole data set.

Data were analysed as follows:

1. Data were audio-recoded and professionally transcribed verbatim. The raw-audio recordings will be listened to a number of times to increase familiarity with the data.
2. Following transcription, the transcripts were read and re-read, initial analytical notes were made, and initial thoughts and impressions documented.
3. The first three transcripts were then analysed and coded, line by line. Using the thematic analysis, ground-up (exploratory) coding guidelines in Braun & Clarke (2006), transcripts were broken down into relevant data extracts, and each of these extracts were be assigned a code. The coding at this stage aimed to classify all of the relevant data extracts, allowing it to be compared in a systematic way with other extracts from the data set. Two researchers carried out this task independently to identify areas of disagreement and to identify reliable coding labels across the extracts. The coded data extracts were then grouped into initial themes and subthemes.
4. The researchers then discussed the codes identified and used these to develop the framework, which was used to code the remaining transcripts. The previously identified themes and subthemes were put into diagrammatic form to aid understanding and comprehension across the research team, clearly defining the themes and subthemes.
5. The remaining transcripts were coded using the identified themes and subthemes.
6. The analysis output was read and the raw transcripts re-read to identify whether the analysis was a true representation of the data. Interpretations of the analysis output were discussed among the team and the key characteristics, themes, and differences identified.
